# Efficacy of Alfacalcidol on PEG-IFN/ Ribavirin Combination Therapy for Elderly Patients With Chronic Hepatitis C: A Pilot Study

**DOI:** 10.5812/hepatmon.14872

**Published:** 2013-12-23

**Authors:** Masanori Atsukawa, Akihito Tsubota, Noritomo Shimada, Chisa Kondo, Norio Itokawa, Ai Nakagawa, Satomi Hashimoto, Takeshi Fukuda, Yoko Matsushita, Hideko Kidokoro, Yoshiyuki Narahara, Katsuhisa Nakatsuka, Katsuhiko Iwakiri, Chiaki Kawamoto, Choitsu Sakamoto

**Affiliations:** 1 Division of Gastroenterology, Department of Internal Medicine, Nippon Medical School Chiba Hokusoh Hospital, Inzai, Chiba, Japan; 2 Institute of Clinical Medicine and Research (ICMR), Jikei University School of Medicine, Kashiwa, Chiba, Japan; 3 Division of Gastroenterology and Hepatology, Shinmatsudo Central General Hospital, Matsudo, Chiba, Japan

**Keywords:** 1-hydroxycholecalciferol, Vitamin D, Ribavirin, Aged, Hepatitis C, Chronic

## Abstract

**Background:**

Serum vitamin D concentration is reported to show a decrease in older age. Patients with chronic hepatitis C (CHC) in Japan are older on average than those in Western countries. Moreover, the outcome of pegylated-interferon (PEG-IFN)/ ribavirin therapy combined with vitamin D in elderly patients is unclear.

**Objectives:**

This pilot study explored the efficacy and safety of alfacalcidol as vitamin D source in PEG-IFN/ ribavirin combination therapy for elderly CHC patients infected with hepatitis C virus genotype 1b.

**Patients and Methods:**

Consecutive twenty CHC patients aged ≥ 65 years were enrolled in this pilot study. Fifteen patients met the inclusion criteria and received PEG-IFN/ ribavirin therapy combined with alfacalcidol. Four-week lead-in of oral alfacalcidol was conducted, and it was subsequently and concurrently administered in PEG-IFN/ ribavirin combination therapy (vitamin D group). Age, gender, and IL28B genotype-matched patients, who received PEG-IFN/ ribavirin alone, were saved as control group (n = 15) to compare the treatment outcome with the vitamin D group.

**Results:**

Subjects consisted of 14 males and 16 females, with a median age of 70 years (65-78). The serum 25 (OH) D3 concentration in females (20 ng/ml, 11-37) was significantly lower than males (27 ng/mL, 13-49) (P = 0.004). Sustained virological response (SVR) rates were 33.3% (5/15) in the control group and 80.0% (12/15) in the vitamin D group, respectively (P = 0.025). While no significant difference was shown in the (SVR) rate between the two groups among males (P = 0.592), in females the SVR rate was significantly higher in the vitamin D group (87.5%, 7/8) than the control group (25.0%, 2/8) (P = 0.041). The relapse rates in the groups with and without alfacalcidol were 7.7% (1/13) and 61.5% (8/13), respectively (P = 0.011). Interestingly, in females, the relapse in the control group was shown in 5 of 7 (71.4%), whereas in the vitamin D group the relapse rate was decreased (1/8, 12.5%) (P = 0.041). No specific adverse events were observed in the vitamin D group.

**Conclusions:**

PEG-IFN/ ribavirin combined with alfacalcidol may be effective and safe in elderly CHC patients. In particular, concomitant administration of alfacalcidol may lead to a reduced relapse rate, and consequently improving the SVR rate in elderly females.

## 1. Background

Therapy for patients with chronic hepatitis C infected with high-viral-load hepatitis C virus (HCV) genotype 1b, which is difficult to treat, has progressed rapidly in the recent years (1, 2). The current standard therapy is pegylated-interferon (PEG-IFN)/ribavirin-based therapy in combination with first-generation protease inhibitors, such as telaprevir and boceprevir. The triple combination treatment increases the rate of sustained virological response (SVR) from 45%–50% to approximately 70% (3-7). However, some patients remain uncured, and adverse effects may occur more frequently and severely using protease inhibitors, prompting further improvement in the therapeutic modalities. Particularly in the elderly patients, the use of such protease inhibitors must be carefully evaluated, because adverse events may become more serious.

Next-generation protease inhibitors that cause adverse events less frequently (8, 9) and therapeutic modalities using a combination of direct antiviral agents alone without IFN (10) are expected to be developed. Young patients at low risk of developing hepatocellular carcinoma can wait until the approval of the next-generation therapeutic modalities. In contrast, elderly patients at high risk of developing hepatocellular carcinoma urgently require new antiviral modalities (11, 12).

The activation of innate and acquired immunity is important for the elimination of the hepatitis C virus. Vitamin D is closely associated with host immunity. Vitamin D enhances the antigen-presenting capacity of dendritic cells, promotes their phagocytic activity toward exogenous antigens (13), and increases the cytotoxic activity of natural killer (NK) cells (14). In vitamin D-deficient mice, abnormal macrophage differentiation is observed (15). Macrophages express vitamin D receptors in the innate immune system (16). Furthermore, mature T-cells and B-cells, which are components of the acquired immune system, also express vitamin D receptors (16), and T-cell receptor signaling is controlled by 1alpha, 25 (OH) 2D3 via receptors (17).

A clinical study reported that the combination of vitamin D supplementation with PEG-IFN/ ribavirin therapy improved the SVR rate in patients with chronic hepatitis C (18). Another study showed that patients with low serum vitamin D levels had a decreased likelihood of achieving SVR in response to PEG-IFN/ ribavirin therapy (19). Furthermore, it has been reported that treatment outcomes could be significantly influenced by serum vitamin D levels and single nucleotide polymorphisms (SNPs) near the interleukin 28B (IL28B) gene (20), which is the strongest predictor of response to PEG-IFN/ ribavirin therapy for patients with chronic hepatitis C of genotype 1 (21-23).

It is conceivable that serum vitamin D levels are lower in the elderly compared to the young patients. Serum vitamin D concentration is reported to show a decrease in older age, particularly in elderly women (19). Patients with chronic hepatitis C in Japan are older on average than those in Western countries. Moreover, the outcome of PEG-IFN/ ribavirin therapy combined with vitamin D in elderly patients is unclear. The present study therefore focused on elderly patients with chronic hepatitis C aged 65 years and older in analyzing the association between serum vitamin D levels and successful therapy. 

Alfacalcidol [1alpha (OH) D3] as a vitamin D supplement is directly metabolized into its active form 1alpha, 25 (OH) 2D3, and is metabolized in the liver [25(OH) ase].

## 2. Objectives

We administered alfacalcidol as vitamin D source in combination with PEG-IFN/ ribavirin therapy for elderly patients with chronic hepatitis C infection of high-viral-load HCV genotype 1b, and compared therapeutic outcome and safety with patients matched for age, gender and IL28B genotype, who received PEG-IFN/ ribavirin alone without alfacalcidol.

## 3. Patients and Methods

### 3.1. Study Design

Pilot study explored the efficacy and safety of PEG-IFN/ ribavirin combination therapy with alfacalcidol for elderly patients with chronic hepatitis C infection of genotype 1b. The inclusion criteria were as follows: age of 65-80 years, high viral load (> 5.0 log IU per mL) by quantitative analysis of HCV-RNA with real-time polymerase chain reaction (PCR), white blood cell count > 2500 per mm3, platelet count > 80000 per mm3, and hemoglobin level > 12 g per dL in laboratory tests. The exclusion criteria were as follows: positive results for hepatitis B surface antigen and antibody to human immunodeficiency virus type-1 (HIV-1), other liver diseases, including autoimmune hepatitis, primary biliary cirrhosis and alcoholic liver disease, liver cirrhosis, current development of hepatocellular carcinoma, severe renal dysfunction, abnormal thyroid function, abnormal parathyroid function, hypercalcemia, poorly controlled diabetes and hypertension, medication with Chinese herbal medicine, history of interstitial pneumonia, severe depression, and allergy to PEG-IFN, ribavirin, alfacalcidol. 

**Figure 1.**
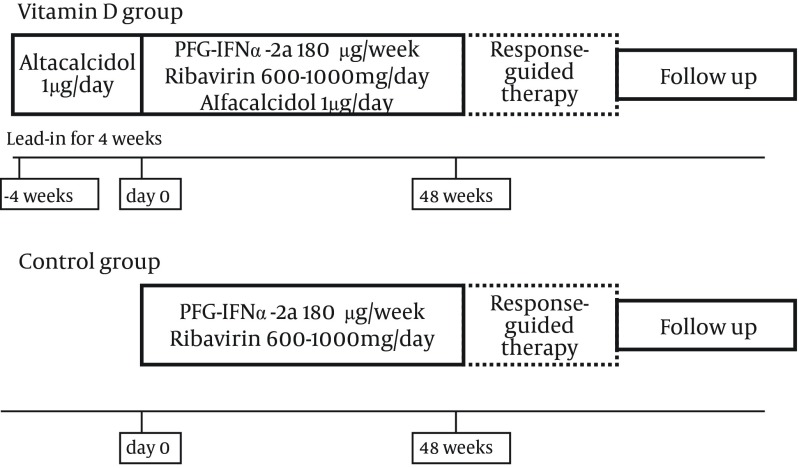
Treatment Protocol

Consecutive twenty patients with chronic hepatitis C of genotype 1b and high-viral-load aged > 65 years visited Nippon Medical School Chiba Hokusoh Hospital between January 2011 and December 2011. Five patients were excluded; 2 cirrhosis, one current development of hepatocellular carcinoma, and two rejected receiving alfacalcidol. Thus, 15 patients met the inclusion criteria and agreed to receive PEG-IFN/ ribavirin therapy combined with alfacalcidol (Table 1 and Figure 1). 

**Table 1.** Baseline Characteristics of Patients in Each Group ^a^

Factors	Vitamin D Group ^b^	Control Group ^b^	P value
**Prior IFN ^b^ mono therapy response naive/relapse/non-virological response**	11/2/2	13/1/1	0.686
**White blood cell count, /mm** ^**3**^	4760 (2650-6530)	4750 (2820-7170)	0.767
**Hemoglobin, g/dL**	13.4 (12.4-14.5)	14.1 (12.3-15.3)	0.128
**Platelet count, ×10^3^/μL**	143 (92-223)	149 (82-227)	0.814
**AST ** ^**b**^ **, IU/L**	59 (20-159)	41 (19-95)	0.280
**ALT ** ^**b**^ **, IU/L**	48 (19-260)	39 (20-149)	0.846
**γGTP , IU/L** ^**b**^ **, IU/L**	33 (13-102)	33 (14-165)	0.927
**T-BIL ** ^**b**^ **, mg/dL**	0.5 (0.3-0.8)	0.6 (0.3-1.6)	0.237
**LDL-C ** ^**b**^ **, mg/dL**	93 (49-153)	103 (53-158)	0.361
**Serum Albumin, g/dL**	4.1 (3.1-4.6)	4.2 (3.5-5.0)	0.260
**AFP ** ^**b**^ **, ng/mL**	4.6 (2.0-130.3)	4.9 (1.6-130.3)	> 0.999
**Prothrombin Time, %**	89.4 (68.8-108.4)	96.7 (68.8-120.3)	0.134
**25 (OH) D3 ng/mL**	22 (11-37)	25 (11-40)	0.751
**1alpha, 25 (OH) 2D3, pg/mL**	63 (36-135)	63 (23-97)	0.480
**HCV-RNA, Log IU/mL**	6.7 (5.1-7.7)	6.6 (5.3-7.3)	0.863
**Fibrosis, F1-2/F3**	12/3	11/4	> 0.999
**ISDR ** ^**b**^ ** mutation 0 or 1/ ≥ 2**	11/4	7/6	0.433
**Core aa 70 Wild/Mutant** ^**b**^	11/4	10/3	> 0.999
**Core aa 91 Wild/ Mutant**	11/4	8/5	0.410
**IL28B (rs8099917) TT/ nonTT** ^**b**^	12/3	13/2	> 0.999

^a^Categorical values are represented as the number of patients. Continuous variables are represented as median (range). Relapse was defined as achievement of the end of treatment response and reappearance of HCV RNA after IFN mono-therapy. Patients who failed to achieve HCV-RNA negativity by the end of prior IFN mono-therapy were considered as non-virological response.

^b^ Abbreviations: PEG-IFN, pegylated-interferon; AST, aspartate aminotransferase; ALT, alanine aminotransferase; LDL-C, low-density lipoprotein cholesterol; γ-GTP, gamma-glutamyltransferase; T-Bil, total bilirubin; ISDR, interferon sensitivity determining region; aa, amino acid; IL28B SNP, Interleukin 28B single nucleotide polymorphism; AFP, alpha-fetoprotein; Vitamin D group, PEG-IFN/ ribavirin/ alfacalcidol; Control group, PEG-IFN/ ribavirin.

Age, gender and IL28B genotype-matched patients, who received PEG-IFN/ ribavirin alone without alfacalcidol, were recruited as the control group to compare the treatment outcome with the vitamin D group. The control group was composed of 15 patients who received PEG-IFN/ ribavirin combination therapy between January 2009 and December 2010 at Nippon Medical School Chiba Hokusoh Hospital and Shinmatsudo Central General Hospital (Table 1). The study protocol was prepared following the ethical guidelines established in accordance with the 2008 Declaration of Helsinki, and was approved by the Ethics Committee of Nippon Medical School Chiba Hokusoh Hospital (Approval numbers: 523010). All patients provided written informed consent. 

### 3.2. Treatment and Definition of Virological Response

PEG-IFNα-2a (PEGASYS; Chugai, Tokyo, Japan) was injected subcutaneously 180 µg per week and oral administration of ribavirin (COPEGUS; Chugai, Tokyo, Japan). Ribavirin dose was adjusted by body weight (600 mg, 800 mg, and 1000 mg per day for < 60kg, 60kg–80kg, and > 80 kg, respectively) based on the guidelines of the Ministry of Health, Labor and Welfare of Japan. During the treatment course, the doses were reduced appropriately when a potentially fatal adverse event such as anemia occurred. For these 15 patients, alfacalcidol (ALFAROL; Chugai, Tokyo, Japan) was administered orally 1 µg/ day as vitamin D source. In the vitamin D group, alfacalcidol was administered for 4 weeks in advance of the combination therapy (Figure 1). The treatment period was 48 weeks if HCV-RNA was undetectable at 12 weeks after the initiation of treatment and was prolonged to 72 weeks if the HCV-RNA became undetectable at 13 weeks or later. Patients who had undetectable levels of the HCV-RNA at the completion of treatment were defined as having an end-of-treatment response (ETR). SVR was defined as HCV-RNA-undetectable status 24 weeks after the completion of treatment. Patients who exhibited an ETR but had detectable levels of the HCV-RNA, 24 weeks after the completion were considered as relapse. Patients who failed to achieve HCV-RNA negativity by the ETR were considered as non-virological response (NVR).The SVR and relapse rates were compared between the vitamin D and control groups. 

### 3.3. Laboratory Tests

Peripheral blood examination, liver function tests and renal function tests were performed weekly until 8 weeks after the initiation of treatment, and then monthly until 24 weeks after the completion of treatment. Both serum 25 (OH) D3, which stably circulates in the body, and 1alpha, 25 (OH) 2D3, an activated form of vitamin D, were measured as serum vitamin D evaluation. The serum 25 (OH) D3 and 1alpha, 25 (OH) 2D3 were measured two times in vitamin D group: at the start of oral alfacalcidol and PEG-IFN/ ribavirin administration in the vitamin D group, and after the administration of alfacalcidol for 4 weeks. Serum 1alpha, 25 (OH) 2D3 and 25 (OH) D3 concentrations were measured by Double-antibody Radioimmunoassay (RIA2 antibody assay) at a commercial laboratory (SRL Laboratory, Tokyo, Japan). HCV-RNA levels were measured using real-time PCR (COBAS AmpliPrep; Roche Diagnostics, Tokyo, Japan). Amino acid substitutions in the core 70 and 91and NS5A regions (interferon-sensitivity determining region; ISDR) of the HCV genome were determined using the direct sequencing method. Core amino acid at position 70 was defined as wild type (arginine) or mutant type (glutamine or histidine), and core amino acid at position 91 was defined as wild type (leucine) or mutant type (methionine). Amino acid mutations in ISDR were defined as wild type (0, 1) or mutant type (≥ 2). Genomic DNA was extracted from whole blood using a DNA Isolation Kit on a MagNA Pure LC instrument (Roche Diagnostics, Basel, Switzerland). SNPs at rs8099917, which is located in the locus adjacent to the IL28B gene on chromosome 19, were determined by real-time PCR using TaqMan® SNP Genotyping Assays on a 7500 Fast Real-Time PCR System (Applied Biosystems, Foster City, CA, The USA). The rs8099917 genotypes were classified into two categories: T/T (major genotype), and non-T/T (minor genotype: T/G or G/G).

### 3.4. Statistical Analysis

Fisher’s exact test and the Mann-Whitney U-test were performed to compare the baseline characteristics. Fisher’s exact test was performed to compare the SVR and relapse rates between the vitamin D and control groups. To confirm the reliability of results regarding the limited number of cases, SVR and relapse rates were analyzed, adopting the bootstrap method. Wilcoxon Signed Ranks Test was performed to compare variables such as 1alpha, 25 (OH) 2D3 between the baseline values and that after 4 weeks of alfacalcidol. The planned sample size was based on the assumption that the SVR rate would be 30% in the control group, and 60% in the vitamin D group, resulting in a necessary sample size of 38 patients in each group with a two-sided significance level of 5%, and statistical power of 80%. However, we were not able to enroll all the 38 patients in each group required by the statistical calculations. The study was stopped at 20 enrolled patients in the vitamin D group due to the slowness of the enrolment procedure. Statistical analyses were performed using IBM SPSS version 17.0 (IBM Japan, Tokyo, Japan). Bootstrap method was performed using SAS Version 9.2 (SAS Institute Japan). The level of significance was set at P < 0.05. 

## 4. Results

A total of 30 patients with a median age of 70 years (range; 65-78), including 14 males and 16 females, were analyzed. Prior treatment response with IFN mono-therapy was relapse and non-virological response in 3 and 3 patients, respectively. There were no significant differences between the 15 patients received PEG-IFN/ ribavirin with alfacalcidol (vitamin D group), and the 15 patients matched for age, gender and IL28B genotype (rs8099917) received PEG-IFN/ ribavirin alone without alfacalcidol (control group) regarding background factors (Table 1). In addition, there were no significant differences between the control and vitamin D groups after 4weeks of alfacalcidol regarding background factors (Tables 2 and 3). 

**Table 2.** Change in Variable Profile After 4 Weeks of Alfacalcidol 1µg Daily ^a^

Variable (Vitamin D Group)	Pre ^b^	Post ^b^	P value ^c^	Difference
**AST ^b^, IU/L**	59 (20-159)	42 (17-199)	0.301	-16 (± 37)
**ALT ** ^**b**^ **, IU/L**	48 (19-260)	37 (19-171)	0.240	-26 (± 51)
**HCV-RNA, Log IU/mL**	6.7 (5.1-7.7)	6.4 (4.3-7.7)	0.078	-1.1(± 2.3)
**25 (OH) D3, ng/mL**	22 (11-37)	27 (7-36)	0.352	2 (± 6)
**1alpha, 25 (OH) 2D3, pg/mL**	63 (36-135)	67 (30-136)	> 0.999	-11(± 43)

^a^Continuous variables are represented as median (range). Differences are represented as mean (± SD).

^b^ Pre, baseline; post, after 4weeks of alfacalcidol; AST, aspartate aminotransferase; ALT, alanine aminotransferase.

^c^ Wilcoxon Signed Ranks Test was performed to compare variables between baseline value and that after 4 weeks of alfacalcidol.

**Table 3.** Comparison of Vitamin D Concentrations Between the Control and Vitamin D Groups After Four Week of Alfacalcidol at Starting PEG-IFN/Ribavirin Combination Therapy ^a^

Variable	Vitamin D group (post ^b^)	Control group (pre ^b^)	P value ^c^	difference
**25 (OH) D3**	27 (7-36)	25 (11-40)	0.371	2 (± 12)
**1alpha, 25 (OH) 2D3 **	67 (30-136)	63 (23-97)	0.563	0 (± 44)

^a^Continuous variables are represented as median (range). Differences are represented as mean (± SD).

^b^ Pre, baseline; post, after 4weeks of alfacalcidol

^c^ Mann-Whitney Test was performed to compare variables between two groups.

Serum vitamin D concentration was measured in the 30 study patients. Serum 25 (OH) D3 was 27 (13-40) ng/mL in males, and 20 (11-37) ng/mL in female (P = 0.004). Thus, the serum 25 (OH) D3 concentrations were significantly lower in females.

**Figure 2.**
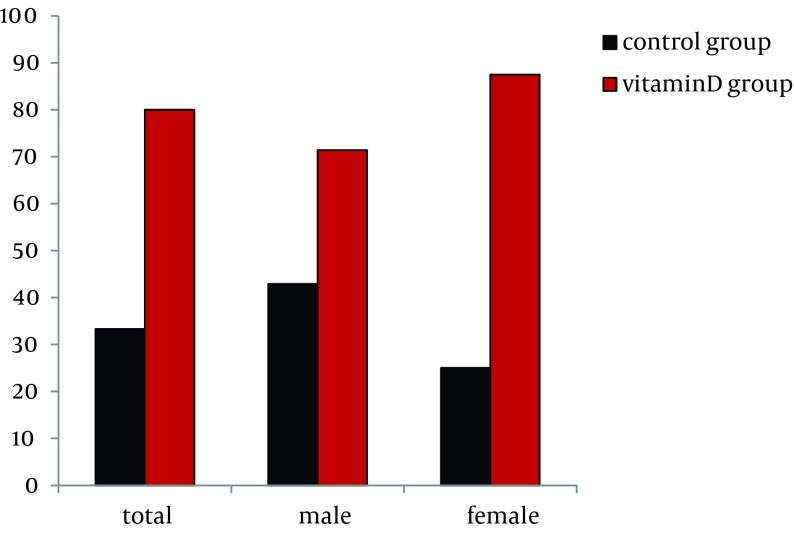
Comparison of the Rate of SVR Between Control Group and Vitamin D Group The results were analyzed by the Fisher’s exact test, adopting the bootstrap method (10,000 times).

The SVR rates in the vitamin D and control groups were 80.0% (12/15) versus 33.3% (5/15) (P = 0.012, bootstrap method), respectively (Figure 2). Five (33.3%) control group patients discontinued treatment and were included in the analysis (intention-to-treat analysis). The reasons for discontinuance were: depression in 1, anorexia in 2, and poor response to treatment (NVR) in 2. Two vitamin D group patients who showed NVR to previous combination treatment discontinued due to poor response to treatment (NVR) regardless of the addition of alfacalcidol. No adverse events due to the addition of alfacalcidol were observed in the vitamin D group. Adverse events such as anemia, neutropenia, skin rash and eruption, gastrointestinal disorders including nausea and anorexia, and psychiatric disorders including insomnia were similar and mild between the two groups. Next, 13 patients in the vitamin D group and 10 patients in the control group completed the therapy as scheduled and were subjected to analysis (per protocol). The SVR rate was numerically higher (92.3%, 12/13) in the vitamin D group than the control group (50.0%, 5/10) (P = 0.055). 

In the male patients, the rate of SVR in the vitamin D group was numerically higher than the control group, but the difference was not statistically significant (P = 0.415, bootstrap method) (Figure 2). Interestingly, in the females, the SVR rate was significantly higher in the vitamin D group (87.5%, 7/8) than the control group (25.0%, 2/8) (P = 0.020, bootstrap method) (Figure 2). 

**Figure 3.**
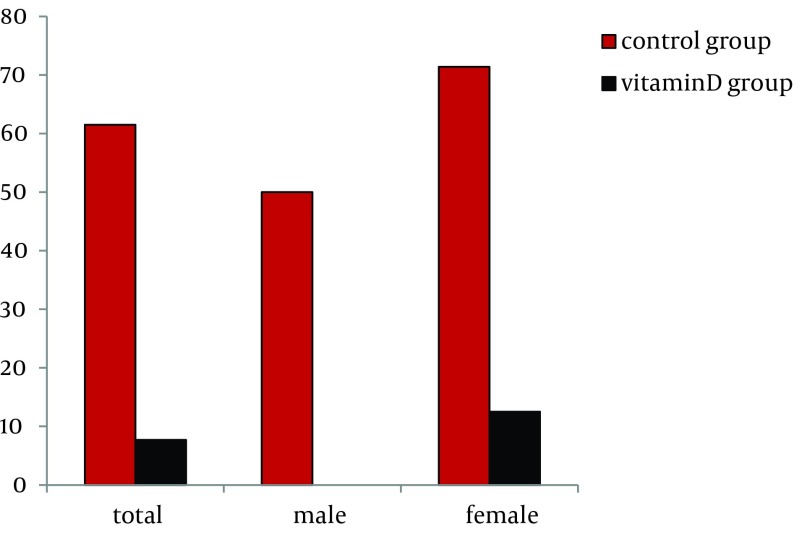
Comparison of the Rate of Relapse Between Control Group and Vitamin D Group The results were analyzed by the Fisher’s exact test, adopting the bootstrap method (10,000 times).

In the control group, relapse was shown after obtaining ETR in 8 patients, including 5 women (62.5%). The median serum 25 (OH) D3 concentration in these patients with relapse was 18 (11-29) ng/mL, lower than the median concentration for all patients. In the vitamin D group treated with alfacalcidol supplementation, a significant decrease was shown in the relapse rate after ETR had been obtained (7.7% vs. 61.5%, P = 0.004, bootstrap method) (Figure 3). Especially, in females, the relapse in the control group was shown in 71.4% (5/7), whereas in the vitamin D group the relapse rate was decreased in 12.5% (1/8) (P = 0.030, bootstrap method) (Figure 3). 

In the vitamin D group, serum 1alpha, 25 (OH) 2D3 concentration numerically increased after administration of alfacalcidol for 4weeks, although the increase was not statistically significant (63.0 pg/mL and 67.0 pg/mL, respectively; P > 0.999). The lead-in duration of 4 weeks may be too short to observe the elevation of serum 1alpha, 25 (OH) 2D3 concentration (Table 2). Moreover, serum 25 (OH) D3 concentration did not apparently increase (22.0 ng/mL and 27.0 ng/mL, respectively; P = 0.352). One possible explanation for this finding is that alfacalcidol is directly metabolized into its active form 1alpha, 25 (OH) 2D3 in the liver (Table 2). 

The rates of total adherence to PEG-IFN were not significantly different between the vitamin D (mean 95.4%, range 70%–100%) and control groups (95.8%, 60%–100%) (P = 0.427). The median dose of total adherence to ribavirin was 8.95 mg/kg/day (range, 3.95–13.04 mg/kg/day) in the vitamin D group, and 9.05 mg/kg/day (3.92–12.31 mg/kg/day) in the control group (P > 0.999).

## 5. Discussion

The present study is the first to analyze the effects of PEG-IFN/ ribavirin therapy combined with alfacalcidol as vitamin D source on elderly patients with chronic hepatitis C infection of high-viral-load genotype 1b. Although there have been some reports on the outcome of combination therapy with vitamin D (18, 24), our report focuses on the treatment response of elderly subjects, who were 65-78 years of age (median, 70 years). Moreover, in other studies patients were given vitamin D3 as vitamin D supplementation, in this study patients were given alfacalcidol [1alpha (OH)D3] as vitamin D source. Alfacalcidol is metabolized rapidly to 1, 25(OH) 2D3 in the liver. 1, 25 (OH) 2D3 is known to be the active form of vitamin D3 and mediates biological activities. 

Elderly patients are likely to have low serum vitamin D levels caused by aging and long disease duration. In fact, serum vitamin D levels have been reported to decrease with age and the progression of liver fibrosis (19). Holick et al. defined ≥ 30 ng/mL of serum 25 (OH) D3 as sufficiency, 21–29 ng/mL as insufficiency, and ≤ 20 ng/mL as deficiency (25). In the present study, the serum 25 (OH) D3 level was deficient or insufficient in 86.7% of the study elderly patients with chronic hepatitis C according to this definition, and sufficient in only a small percentage (13.3%).

Several recent reports have documented the direct anti-HCV effects of vitamin D. In an in vitro study using Huh7.5 cells, the addition of vitamin D and its metabolite 1α, 25 (OH) 2D3 reduced secretion of HCV from hepatocytes into the cell medium; this effect was synergistically enhanced by IFN administration (26). In another in vitro study using HCV-JFH1, 25 (OH) D3 decreased the amount of HCV core protein by suppressing the formation of HCV particles (27).

The results of this study suggest that the addition of alfacalcidol could potentially suppress relapse in patients with low serum vitamin D concentration, particularly elderly females. Since treatment-induced anemia occurs more frequently and more severely in the elderly than younger people (28), the total dose of ribavirin may be reduced in elderly subjects. As mentioned earlier, the reduction of total dose of ribavirin is an important factor contributing to viral relapse after the completion of PEG-IFN/ribavirin therapy (29). The results suggest that the addition of alfacalcidol as vitamin D source may potentially improve the therapeutic outcome of such elderly patients who showed relapse after previous combination treatment due to reduced drug adherence.

This study also showed that alfacalcidol combination therapy had some limitations. In elderly patients with the difficult-to-treat IL28B SNP minor genotype or who showed NVR to previous combination treatment, even the addition of vitamin D had little effect on the improvement of the SVR rate. Two patients with pretreatment non-response by IFN therapy in the vitamin D group also showed non-virological response. Moreover, SVR rate among patients with IL28B minor genotype in the vitamin D group was 33.3% (1 of 3). Alternatively, long-term IFN therapy with low doses is recommended to prevent the development of hepatocellular carcinoma (30-32).

As for gender difference, the average serum 25 (OH) D3 concentration of female elderly patients was significantly lower than male elderly patients (median; 20 ng/mL versus 27 ng/mL) in this study. It has been reported that females of age 55 years and over have lower serum 25 (OH) D3 concentration than females younger than 55 years, and males do not differ in serum 25 (OH) D3 concentration between the two age groups (19). The addition of vitamin D may be beneficial to female elderly patients with low serum vitamin D concentration, because Japanese elderly patients have poor response to the conventional combination therapy (33, 34). By extension, the addition of vitamin D for patients with low serum vitamin D concentration is expected to further improving the outcome of PEG-IFN/ ribavirin therapy in combination with protease inhibitors such as telaprevir.

The present study had some limitations; these included a small number of subjects and lack of a randomized controlled design. Although sample size was too small, patients were matched for gender, age and IL28B genotype for the control group. As a result, strong predictors for PEG-IFN/ribavirin combination therapy such as IL28B genotype (21-23) and core amino acid 70 substitutions (35) were similar in the two groups. Moreover, analysis was performed, adopting the bootstrap method. Next, the serum vitamin D concentration was measured at the start of therapy in various seasons. It has been reported that the serum vitamin D concentration is higher in summer and autumn than winter and spring.

In this study, alfacalcidol was orally administered at 1 µg/day as a vitamin D supplement. Since it is directly metabolized into its active form 1alpha, 25 (OH) 2D3 in the liver, 25(OH) D3 levels do not increase theoretically (36), as also described in this study. Therefore, the level of 1alpha, 25 (OH) 2D3 in the liver may be an important factor in the achievement of SVR, although the serum concentration was not significant in this study. In fact, it has been pointed out that 1alpha, 25 (OH) 2D3 is important for the suppression of HCV (26). However, we did not confirm whether this dose was sufficient for the combination therapy. In this stratified analysis, serum 25 (OH) D3 concentration might be a useful indicator to predict the SVR and relapse rates. The reason may be that the half-life of serum 25 (OH) D3 is longer, hence more stable than 1alpha, 25 (OH) 2D3, and consequently maintains a high concentration in serum. Accordingly, further investigation is required to examine whether vitamin D or its metabolites exert efficacy in vivo, because there is no clinical study comparing the efficacy among vitamin D forms and its metabolites.

In conclusion, the present study demonstrated that PEG-IFN/ ribavirin therapy combined with alfacalcidol may be effective and safe in elderly patients with chronic hepatitis C of high-viral-load HCV genotype 1b. In particular, female elderly patients with low serum vitamin D concentration, who are less likely to respond virologically to PEG-IFN/ ribavirin, may benefit from the combination of PEG-IFN/ ribavirin and alfacalcidol through its effect in reducing the relapse rate and consequently improving the SVR rate.
